# Fluency Is Good, but Comprehension Is Better: The Impact of Fluency and Comprehension on Mathematical Word Problem Solving

**DOI:** 10.1002/dys.70031

**Published:** 2026-03-16

**Authors:** Anke Treutlein

**Affiliations:** ^1^ Department of Education University of Stuttgart Stuttgart Germany

**Keywords:** comprehension, dyslexia, fluency, large‐scale assessment, mathematics, word problems

## Abstract

The impact of fluency and comprehension on mathematical word problem solving is explored using data on fifth‐graders who took part in the German National Educational Panel Study (NEPS). The Multiple Deficit Model (MDM) suggests that the impact of fluency and comprehension on mathematical word problem solving should be the same for students with and without dyslexia, even if they are at different levels in reading and mathematics. This study shows that students with poor fluency or comprehension score lower in word problems than typical readers. For all students with reading difficulties, the impact of fluency on mathematical performance is less than it is for typical readers. For students with deficits in either comprehension or fluency alone, the impact of comprehension on word problem solving is the same as for typical readers. But the impact of comprehension is less if the students are weak in both fluency and comprehension, which, according to the MDM, suggests the presence of additional or different underlying cognitive deficits. While students with poor fluency can compensate for this deficit with reading comprehension, students with poor comprehension cannot compensate with fluency. Comprehension has a larger impact on mathematical word problem solving than fluency.

## Practitioner Points

- Fluency and comprehension impact mathematical word problem solving.
- Comprehension has a larger impact on word problem solving than fluency.
- Early diagnosis of dyslexia is essential to obtain appropriate treatment.

## 1 Introduction

Dyslexia, or specific reading disorder, is the most common learning disorder, affecting an estimated 5%–15% of the world's population (International Dyslexia Association 2020; Schulte‐Körne 2014; Siegel 2006). According to current definitions, dyslexia is characterised by significant difficulties in reading accuracy, fluency (speed) and comprehension that cannot be better explained by intellectual disabilities.^1^

In school settings, difficulties in reading accuracy, fluency and comprehension become apparent when students diagnosed with dyslexia are required to work with written tasks. Typical challenges include difficulties understanding instructions, slow reading and missing important information due to inaccurate decoding. These problems are not attributable to limited cognitive ability or insufficient domain knowledge, but occur whenever tasks rely on written text. The challenge is particularly pronounced in mathematical word problems.

Although previous research has examined the role of reading accuracy and comprehension in mathematical word problem solving, the impact of fluency has not yet been systematically investigated. This study addresses this gap by examining how fluency (reading speed) relates to mathematical achievement in word problems in students with dyslexia and typical readers. Building on existing theoretical and empirical evidence, I hypothesise that fluency and comprehension exert a stronger influence on mathematical competence in students with dyslexia than in typical readers when mathematical competence is measured using word problems.

### 1.1 The Multiple Deficit Model (MDM) as Framework for Understanding Learning Disabilities

A learning disorder can be conceptualised as the lower tail of a normal distribution of an ability. As Vanbinst et al. (2020, 145) argue, ‘findings from studies focusing on the lower tail or hence disability, can be extended to the entire distribution of individual differences, and interestingly, also the other way around’. Accordingly, they propose the use of the MDM to study not only the lower tail of an ability distribution but also the full range of individual differences.

The MDM emphasises that dyslexia is not caused by a single underlying cognitive deficit. Instead, multiple factors contribute to its manifestation, with disorders like dyslexia or dyscalculia emerging from the interaction of multiple risk and protective factors (Pennington 2006; van Bergen et al. 2014).

According to the MDM, learning disabilities can share underlying cognitive deficits that affect multiple domains. The model therefore offers a coherent framework for explaining the frequent co‐occurrence of disorders such as dyslexia and dyscalculia. For instance, phonological processing has been identified as a shared cognitive correlate of both reading and arithmetic skills (Jordan et al. 2010; Simmons and Singleton 2008; Singer et al. 2019), whereas semantic skills predict performance in word problem solving and numeration (Boonen et al. 2016; Singer et al. 2019). Furthermore, word problem solving depends on reading comprehension—a skill that is impaired in students with dyslexia. Evidence from twin studies suggests that these associations between reading and mathematics are largely attributable to shared genetic factors rather than direct causal relations (van Bergen et al. 2025).

Shared cognitive deficits are reflected in lower or higher achievements in both reading and mathematics. Building on this perspective, the following sections review studies that examine level differences in reading and mathematical skills in students with or without dyslexia. Importantly, the MDM predicts that the impact of one skill (e. g. phonological skills) on another domain (e.g., mathematics) should be comparable across different levels of the respective skill. Before turning to this literature, however, mathematical word problems need to be defined.

### 1.2 Defining Word Problems

Word problems, also referred to as story problems, are tasks in mathematics that are presented in a linguistic form that requires students to extract relevant information from a text and translate it into mathematical notation. Unlike purely arithmetic problems, solving word problems first requires reading and understanding the textual information before mathematical operations can be applied. Reflecting this emphasis, problem solving is identified as a core objective of mathematics education in many contemporary curricula (e.g., Department of Education 2013; KMK—Ständige Konferenz der Kultusminister in der Bundesrepublik Deutschland 2022).

The characteristics of mathematical word problems can present challenges even for typical readers. It is not surprising that Roe and Taube (2006) conclude:

Students who fail to solve mathematical problems might do so because they are unable to do one, some or all of the following correctly: correctly decode words, understand their exact meaning in a mathematical context, reflect on the mathematical problem, actually solve the problem and present the solution to the problem in written words so that others can understand it (Roe and Taube 2006, 148).

Beyond the ability to decode words (technical reading, e. g., phonological decoding), students require adequate reading comprehension skills to construct a mental representation of a word problem (Fuchs et al. 2006; Kyttälä and Björn 2014; Vilenius‐Tuohimaa et al. 2008). That mental representation of the problem consists of both a situational model and a mathematical model (Boonen et al. 2016, 2013; Kyttälä and Björn 2014; Vilenius‐Tuohimaa et al. 2008).

The construction of a situational model relies on visual‐schematic representations of the problem as well as relational processing, and is, particularly, dependent on reading comprehension (Boonen et al. 2016, 2013). These situational models provide the foundation for a mathematical model, which, in turn, guides learners through the problem‐solving process, assuming that they have the necessary mathematical skills (Kyttälä and Björn 2014).

Students with dyslexia struggle with basic reading skills, such as phonological decoding (the difficulty in reading accuracy mentioned in the DSM‐V) and reading comprehension. For this reason, it can be assumed that they will also struggle with the inherent demands of mathematical word problems. The next section reviews the empirical evidence on the role of reading skills in mathematical performance.

### 1.3 Phonological Decoding and Mathematics

The way cognitive deficits in phonological skills influence both reading and mathematics, as proposed by the MDM, is explained by Dehaene's triple‐code model, which emphasises the role of phonological processing in arithmetic. According to Dehaene (1992), a number is represented in three different codes: a verbal code, an Arabic code and an analogue magnitude representation. Each code can be translated into one of the other two. Dehaene (1992) suggests that arithmetic facts are stored as verbal associations. He links the verbal code to arithmetic fact retrieval. Because the verbal codes are used during arithmetic tasks, deficits in phonological processing will impair this process and influence arithmetic achievement.

A large number of studies on level differences support this assumption. Simmons and Singleton (2008) reviewed studies of the role of phonological processing in students with dyslexia in arithmetic. They concluded that reading difficulties and fact retrieval share the same underlying deficit in phonological processing. They also proposed three causal links between weak phonological processing and aspects of mathematics that involve the manipulation of verbal codes. First, verbal codes are used to build up a store of arithmetic facts. Deficits in phonological representations impair the development of this store of facts because the phonological loop is not functioning properly (see also Krajewski and Schneider 2009). Second, once the store of arithmetic facts is built up, access to the phonological codes therein is slow. The third link is the assumption that the neurological basis for dealing with phonological representations is impaired in students with dyslexia (Simmons and Singleton 2008). The difficulties of students with dyslexia in arithmetic, therefore, are explained by the phonological deficit that characterises dyslexia.

These same difficulties become apparent in the slow counting and slow fact retrieval of students with dyslexia. Dyslexic students with otherwise normal mathematics achievement performed worse than controls in single‐digit arithmetic (Boets and de Smedt 2010). Similar deficits were reported for third‐, fourth‐ and fifth‐graders (Clercq‐Quaegebeur de et al. 2018; Vilenius‐Tuohimaa et al. 2008; Vukovic et al. 2010) and for eighth‐graders (Kyttälä and Björn 2014).

Further confirmation of Dehaene's model can be seen in Göbel and Snowling (2010), who studied adults with and without dyslexia. They found that difficulties in arithmetic in individuals with dyslexia are restricted to the verbal code. This result is in line with the triple code model. Träff and Passolunghi (2015) obtained a similar result in a study of third‐ and fourth‐grade students with and without dyslexia. They found that learners with dyslexia performed worse in word‐problem solving, but these difficulties disappeared when controlling for phonological skills. In their study, all of the word problems were read out loud to the participants. This indicates that poor word‐decoding skills used in reading word problems were not the cause of the difficulties. Instead, it suggests that phonological skills contribute to word problem solving (Träff and Passolunghi 2015).

All of the studies discussed above focused on level differences in reading and mathematics, comparing the lower tail of the reading distribution (learners with dyslexia) to the rest (controls). Fuchs et al. (2006) studied third‐graders and showed in a path model that phonological decoding predicts arithmetic skills and also—moderated by these arithmetic skills—arithmetic word problems. But it does not predict algorithmic computation. This is in line with Dehaene's assumption that phonological processing does not affect every mathematical task.

According to the MDM, the level differences could be explained by a shared cognitive deficit between phonological decoding and arithmetic or fact retrieval. We do not know, however, if phonological decoding affects arithmetic to the same degree for learners with dyslexia as it does for controls.

### 1.4 Reading Comprehension and Mathematics

Studies (e.g., Boonen et al. 2013; Vilenius‐Tuohimaa et al. 2008) have shown that reading comprehension facilitates the construction of an adequate situational model and word problem solving. This indirect path between reading comprehension and word problem solving via relational processing could point to a shared cognitive skill. Reading comprehension, however, also predicts word problem solving skills directly (Boonen et al. 2013). This is especially true of semantically complex word problems:

word problems containing semantically complex features require both accurate mental representation skills and reading comprehension skills, whereas for word problems with a lower semantic‐linguistic complexity, well‐developed mental representational skills might be sufficient (Boonen et al. 2016, 2).

Another study of fourth‐graders also showed that reading comprehension predicts word‐problem solving (Vilenius‐Tuohimaa et al. 2008). Moreover, decoding skills influence both reading comprehension and word problem solving. Similar results were reported for eighth‐grade students (Kyttälä and Björn 2014): Decoding skills predicted calculation performance, whereas reading comprehension did not. Furthermore, both decoding and reading comprehension predicted word‐problem performance. In younger children—Year 2 students—word problem solving was uniquely associated with reading comprehension (Bjork and Bowyer‐Crane 2013).

None of the studies mentioned above explicitly tested the impact of reading comprehension on word problem solving in different levels of reading comprehension (e. g. students with and without dyslexia). Other studies have examined children with different deficits in reading and mathematics. For example, Jordan et al. (2003) examined four groups of students: those with math difficulties only, reading difficulties only, both math‐ and reading‐difficulty and typical achievement. They found that the comorbid group performed worse on word‐problem solving than the other groups. The comorbid group also showed poorer number‐fact retrieval than typical or reading‐only students, though it was no worse than the math‐only group. Consequently, the authors hypothesised that strengths in one domain may compensate for weaknesses in the other when solving word problems (Jordan et al. 2003). Students with difficulties in both areas, however, are not able to compensate in this way.

Pimperton and Nation (2010) examined students with difficulties in reading comprehension but not in phonological decoding or sight word reading. They found that students with poor comprehension perform as well as the control group in numerical operations tasks, but are worse in mathematical reasoning tasks that are word problems. Similar results were obtained by Vukovic et al. (2010), who examined third‐graders with reading‐comprehension difficulties alone or with additional phonological‐processing deficits. The former group performed as well as controls and outperformed the latter group on arithmetic‐fact fluency and operations tasks. In word problem solving, however, both reading‐difficulty groups scored lower than controls. These results support the assumption that phonological processing is the underlying deficit in reading and mathematical problems.

To summarise, the current state of research suggests that reading comprehension is linked to successful word problem solving. It is, particularly, noteworthy that in all of the studies mentioned above, the word problems were read out loud to the participants. Even then, reading comprehension still played an important role. It is not clear, however, if reading comprehension has a similar impact on word problem solving for different levels of reading comprehension, as the MDM would assume.

### 1.5 Fluency and Mathematics

According to well‐established models of reading components and reading development, fluency develops as phonological processing becomes more automatic (Hoover and Gough 1990; Kim et al. 2012; Klauda and Guthrie 2008; LaBerge and Samuels 1974). Early readers have to switch their attention between decoding, phonological processing and understanding the text. Fluent readers read words automatically and therefore do not have to switch, but can focus solely on comprehension (LaBerge and Samuels 1974). With fluency, the cognitive load of phonological decoding decreases and more cognitive capacity can be used for understanding the text. Fluency is seen as necessary for reading comprehension (Hoover and Gough 1990; Kim et al. 2012).

Fluency is another basic reading skill that is often impaired in persons with dyslexia. In German learners with dyslexia, the deficit in fluency is pervasive and stable, even if reading accuracy is high (Wimmer 1993). Fluency can be seen as a form of fast word retrieval and demands phonological processing. Thus, deficits in phonological processing affect both fluency and mathematical fact retrieval (Fuchs et al. 2006). Clearly, fluency should have an impact on word problem solving because slow readers read less text in the same amount of time as typical readers. This means that less fluent readers have less time to solve the same number of mathematical problems.

The effect of fluency on word problem solving has yet to be studied. As fluency facilitates reading comprehension (e.g., Klauda and Guthrie 2008), it is reasonable to suspect that it also affects word problem solving.

### 1.6 Research Rationale and Hypotheses of the Present Study

The studies of word problem solving summarised above suggest that word problem solving is influenced by reading comprehension and by phonological processing if fact retrieval is part of the problem solving. Both skills can be impaired in students with dyslexia. In all of the studies presented above, word problems were read out loud to the participants. This attempt to compensate for potential difficulties in reading represents a form of assistance that students with dyslexia do not have in daily life or even in most tests they take at school.

The impact of fluency on word problem solving has not yet been studied. There are, however, theoretical assumptions and empirical findings that suggest that fluency facilitates comprehension. This paper addresses the deficits of past studies and aims to clarify how fluency and comprehension impact word problem solving when word problems are read by the students themselves. Since reading skills have an impact on word problem solving when problems are read out loud, it is possible that the impact is different for students with and without dyslexia. If so, then the discrepancy is not explained by the MDM. The present study aims to clarify the following three questions:

1. When considering level differences, is the mathematical competence of students with dyslexia and typical readers comparable when they are solving word problems?
2. Does fluency have the same impact on mathematical competence in students with dyslexia and typical readers or is mathematical competence influenced more or less strongly in the different groups?
3. Does comprehension have the same impact on mathematical competence in students with dyslexia and typical readers or is mathematical competence influenced more or less strongly in the different groups?

It can be hypothesised that when mathematical competence is assessed with word problems (which are not read out loud), students with dyslexia will perform more poorly than typical readers because of their reading deficit. Consequently, they should score lower in word problems for students with dyslexia. According to the MDM (Vanbinst et al. 2020), however, the impact of reading skills on word problem solving should be the same over the whole distribution of reading if the same underlying cognitive skills affect mathematical achievement.

More specifically, the following results are expected:

1. Students with dyslexia should score lower in word problem solving than students without dyslexia (Pimperton and Nation 2010; Träff and Passolunghi 2015; Vukovic et al. 2010).
2. The impact of fluency on mathematical word problem solving—namely the beta‐weights in regression models—should be lower for students with dyslexia than without dyslexia.
3. The impact of reading comprehension on mathematical word problem solving—namely the beta‐weights in regression models—should be lower for students with than without dyslexia (Jordan et al. 2003; Pimperton and Nation 2010; Vukovic et al. 2010).

## 2 Method

### 2.1 Data

This paper uses data from the NEPS (see Blossfeld and Roßbach 2019). The NEPS is carried out by the Leibniz Institute for Educational Trajectories (LIfBi, Germany) in cooperation with a nationwide network. For this study, data from starting cohort Grade 5 is used (NEPS Network 2021). Grade 5 was chosen as a sample group because it is the first grade after which students have transitioned into a secondary school in Germany. Grade 5 requires students to read more often and with less help than in primary school. Furthermore, both DSM‐V and ICD‐11 describe dyslexia as persistent difficulties. By Grade 5, those difficulties have been present for at least 4 years and, in some cases, remedial measures or support may already be in place. Providing support for students with difficulties in reading is usually seen as a task for the primary school.

The sample for starting cohort Grade 5 consisted of 6112 fifth graders, 5525 of whom attended regular schools and 587 of whom attended special needs schools. Data are available for 5778 fifth graders (for more information on sampling see Skopek et al. 2012). Moreover, the NEPS excluded students enrolled in special schools where teaching is predominantly conducted in a foreign language (Skopek et al. 2012). For more information on the NEPS see Blossfeld et al. (2011). Because data on student competences are only available for students in regular schools, the following analysis focuses only on that sample.

The data examined here were collected in the fall and winter of 2010. The NEPS data comprise a variety of different competence measures in several domains and background information of the students. For the analysis in this paper, measures of non‐verbal cognitive basic skills (reasoning), reading comprehension and fluency were selected. For the analyses, furthermore, data on mathematical competence were used.

### 2.2 Measures

#### 2.2.1 Non‐Verbal Cognitive Basic Skills—Reasoning

Non‐verbal cognitive basic skills were measured in the NEPS by a test of perceptual speed and a test of reasoning (see Haberkorn and Pohl 2013). Because a speeded measure of reading is used in this paper, non‐verbal cognitive basic skills were determined by the reasoning test only. The reasoning test was a classical matrices test with fields that contained geometrical elements following logical rules. The students' task was to determine which element fits best in a free field within the pattern. To do so, they had to identify the logical rules behind the pattern. Three sets, each containing four items, were used in the NEPS. Students were given 3 min for each set. The data show that most students were able to complete the test in that time (Haberkorn and Pohl 2013). This means that the score reflects correctly solved items and that the time allotted was not a factor. On average, fifth‐graders scored 6.89 (SD = 2.62). Reliability is 0.66 (Novita 2020).

For further analyses, scores were transformed into *z*‐scores.

#### 2.2.2 Fluency/Reading Speed

The test to measure fluency consisted of 51 sentences that had to be judged as true or false within 2 min (for more details see Zimmermann et al. 2012). As the test aimed to measure automatized reading processes, all of the sentences could be judged as true or false using common knowledge from daily‐life situations (e. g. ‘There is a bath tub in every garage’). The number of sentences evaluated incorrectly by the students—0.64 sentences on average—shows that the sentences were easy to evaluate. The sentences were so easy to understand that the differences between students are likely the result of different reading speeds and were not caused by different levels of reading comprehension. This might be the reason for the low correlation between fluency and comprehension (see Table 1). A sumscore was calculated by adding up all sentences correctly identified as true or false within the 2 min of testing time. On average, the fifth‐graders in the NEPS‐sample evaluated *M* = 21.24 sentences correctly (SD = 7, see Zimmermann et al. 2012). This shows that not all students could evaluate all sentences within the given time, even if they made few mistakes. Reliability is 0.98 (Wolter and Gehrer 2020).

**TABLE 1:** Inter‐correlations of reasoning, reading tests and mathematical competence.

Variable	Fluency	Mathematical competence	Reading comprehension
Reasoning	0.16**	0.45**	0.33**
Fluency		0.29**	0.30**
Mathematical competence			0.59**

** *p* < 0.01.

For further analyses, scores were transformed into *z*‐scores.

#### 2.2.3 Reading Comprehension

The reading comprehension test consisted of five different text types: informational texts, commenting texts, literary texts, instructional texts and advertising texts. For each text, three different task types were constructed that reflected three cognitive abilities: finding information in the text, drawing text‐related conclusions and reflecting on and assessing the text. The majority of the tasks were in multiple‐choice format. Some of the items were decision‐making tasks and matching tasks. In 28 min, students had to answer 32 items (five to seven items per text). For more details see Gehrer et al. (2012).

The test was scaled based on Item Response Theory. The items showed a good fit, without DIF effects (Pohl et al. 2012). The test measures reading ability especially in low‐performing students (Pohl et al. 2012). With 0.81, the test has a good reliability (Gehrer et al. 2020).

#### 2.2.4 Mathematical Competence

The NEPS concept of mathematical competence is compatible with the concept of mathematical literacy as used in PISA and with the German Mathematics Education Standards. As Neumann et al. (2013, 85) put it, ‘the concept of mathematical competence in NEPS could be described as relevant for future life and, to a limited extent, as curriculum‐based as well’. The concept defines four content areas and six cognitive components (Neumann et al. 2013; Schnittjer and Duchhardt 2015). The situations described in the items were embedded in daily life or typical to the age cohort (Neumann et al. 2013). All items can therefore be categorised as word problems as defined above. Importantly, all students were required to read the word problems by themselves.

The test in Grade 5 consisted of 24 items and was scaled based on Item Response Theory. Scores were transformed to WLEs (weighted likelihood estimates). WLEs are point estimates that reflect the most likely competence score for each student given the responses of that student. WLEs range between −4.70 and 4.03, SE (WLE) between 0.45 and 1.57. For further analyses, WLEs were transformed into z‐scores. Reliability is 0.8 (Petersen et al. 2020).

As can be seen in Table 1, intercorrelations ranged between *r* = 0.16 and *r* = 0.59. The substantial correlation between mathematical competence and reading comprehension points to the underlying conceptualization as literacy (see above).

### 2.3 Groups: Students With Reading Difficulties and Typical Readers

There were no certain diagnoses of dyslexia amongst the participants. Parents were asked if their child was diagnosed with dyslexia, but the parental answer cannot be seen as a valid diagnosis. For this reason, fluency and reading comprehension were used to identify students with reading difficulties. According to the DSM‐V and ICD‐11, reading fluency or comprehension below what is to be expected based on a student's chronological age is one of the criteria for a diagnosis of specific learning disability with impairment in reading (DSM‐V) or developmental learning disorder with impairment in reading (ICD‐11). Reading impairment was operationalised as below average, meaning *T* < 30.^2^ This produced groups of students with below average reading comprehension, below average fluency or both.

Only the discrepancy in what is to be expected based on chronological age was used to identify groups. A discrepancy to intellectual function, which ICD‐11 requires, was not used, as the DSM‐V and the German Leitlinien (recommended procedures) do not require it. To eliminate students with difficulties caused by intellectual disabilities, only students with a *T*‐score in reasoning of 30 and above (equivalent to IQ = 70) were taken into the analysis. All other students were coded as typical readers.

This leaves a final sample of *N* = 5010 students, of whom 3904 were typical readers (77.9%), 526 of them (10.5%) with below average reading comprehension, 352 of them (7.0%) with below average fluency and 228 (4.6%) with both. Means and standard deviations for these groups are presented in Table 2. As can be seen in Table 2, students with reading difficulties scored lower on the reasoning test than the controls. Therefore, reasoning will be used as a covariate in the analyses.

**TABLE 2:** Mean *z*‐scores of reasoning, fluency and reading comprehension in the four groups.

Difficulties in	Mean reasoning (SD)	Mean fluency (SD)	Mean reading comprehension (SD)
Reading comprehension	−0.77 (0.93)	−0.16 (0.85)	−1.44 (0.34)
Fluency	−0.17 (0.96)	−1.33 (0.38)	−0.11 (0.71)
Reading comprehension and fluency	−0.73 (0.88)	−1.40 (0.34)	−1.67 (0.44)
No difficulties	0.16 (0.95)	0.22 (0.91)	0.30 (0.82)

In the first wave of the NEPS, students and parents were asked if the child was diagnosed with dyslexia (Leibniz Institute for Educational Trajectories 2018). Interestingly, many of the parents of children with reading difficulties stated that there was no diagnosis of dyslexia for their child (see Table 3). The probability of having a dyslexia diagnosis is higher when the child has difficulties in both reading comprehension and fluency.

**TABLE 3:** Dyslexia diagnosis according to the parents in the four groups.

Difficulties in	Dyslexia diagnosis according to parents
Reading comprehension	14.3%
Fluency	15.1%
Reading comprehension and fluency	27.1%
No difficulties	4.3%

### 2.4 Analyses

To answer the first research question, whether or not word problem solving by students with reading difficulties is comparable to word problem solving by typical readers, an ANOVA was run. To determine if the mathematical competence of typical readers and children with reading difficulties is impacted by fluency or comprehension differently, two separate regression analyses with interaction terms were performed (moderated regression analysis). Mathematical competence was regressed on fluency or comprehension, reasoning (as a control variable), group and the interactions of group and fluency or group and comprehension, respectively. A single regression analysis is too cumbersome for both reading fluency and comprehension, so two analyses have been employed here and, in any case, yield the same results as the single regression. As mentioned above (Table 2), the groups differ in their reasoning. Therefore, this measure was entered as a control variable.

All predictors were entered simultaneously into the regression model. The mathematical competence, fluency, comprehension and reasoning were entered as *z*‐standardised variables so that coefficients can be interpreted as standardised Beta‐coefficients. Group was entered as three contrast‐coded dummy variables: the first dummy contrasts typical readers (coded as 0.75) and students with reading difficulties (regardless of difficulties in fluency or comprehension, coded as −0.25). The second contrast determines the difference between the two groups of students with reading difficulties in one area. Students with difficulties in fluency only were coded as −1/2 and students with difficulties in comprehension only were coded as 1/2. The third dummy contrasts students with difficulties in fluency and comprehension (coded as −1/2) and students with difficulties in fluency only (coded as 1/2).

## 3 Results

The ANOVA revealed that mathematical competence differs significantly between the groups (*F* (3, 5006) = 454.27; *p* < 0.001). Post hoc tests show that all groups differ significantly in their mathematical competence. Typical readers achieve on average *z* = 0.23, students with difficulties in fluency only on average *z* = −0.28, students with difficulties in comprehension on average *z* = −0.98 and students with difficulties in both areas on average *z* = −1.22.

### 3.1 Impact of Fluency on Mathematical Competence

In a second step, a regression model for the impact of fluency on mathematical competence was built. As can be seen in Table 4, the main effects of reasoning and fluency were significant. In all groups, good reasoning and fast reading are associated with better mathematical competence. Typical readers outperform students with reading difficulties in mathematical competence. All interactions can be classified as significant—fluency seems to have a different impact on students with different difficulties.

**TABLE 4:** Moderated regression analysis for the effects of group and fluency on mathematical competence.

Effect	*B*	SE	95% CI		*t*
			LL	UL	
Intercept	−0.07	0.03	−0.14	−0.007	−2.16*
Reasoning^a^	0.37	0.01	0.35	0.39	31.94***
Fluency^a^	0.28	0.02	0.23	0.32	11.46***
Typical reader vs. students with reading difficulties^b^	0.38	0.04	0.30	0.46	8.90***
Students with difficulties in fluency vs. difficulties in reading comprehension^c^	−1.09	0.07	−1.22	−0.96	−16.39***
Students with difficulties in fluency only vs. difficulties in fluency and comprehension^d^	−0.19	0.10	−0.38	−0.003	−1.99*
Typical readers vs. students with reading difficulties × fluency	−0.17	0.04	−0.24	−0.10	−4.69***
Students with difficulties in fluency vs. difficulties in reading comprehension × fluency	−0.43	0.07	−0.57	−0.29	−5.97***
Students with difficulties in fluency only vs. difficulties in fluency and comprehension × fluency	−0.56	0.07	−0.70	−0.43	−8.17***
Model fit	*R* ^2^ = 0.43				
Omnibus test	*F* (8, 5009) = 462.90, *p* < 0.001				

Abbreviations: CI = confidence interval, LL = lower limit, UL = upper limit.

^a^ z‐Standardised. Coefficients can be interpreted like standardised betas.

^b^ Contrast‐coded: typical readers = 0.75, students with reading difficulties = −0.25.

^c^ Contrast‐coded: students with difficulties in fluency only = −1/2, students with difficulties in reading comprehension = 1/2.

^d^ Contrast‐coded: students with difficulties in fluency only = 1/2, students with difficulties in reading comprehension and fluency = −1/2.

* *p* < 0.05.

*** *p* < 0.001.

**FIGURE 1:**
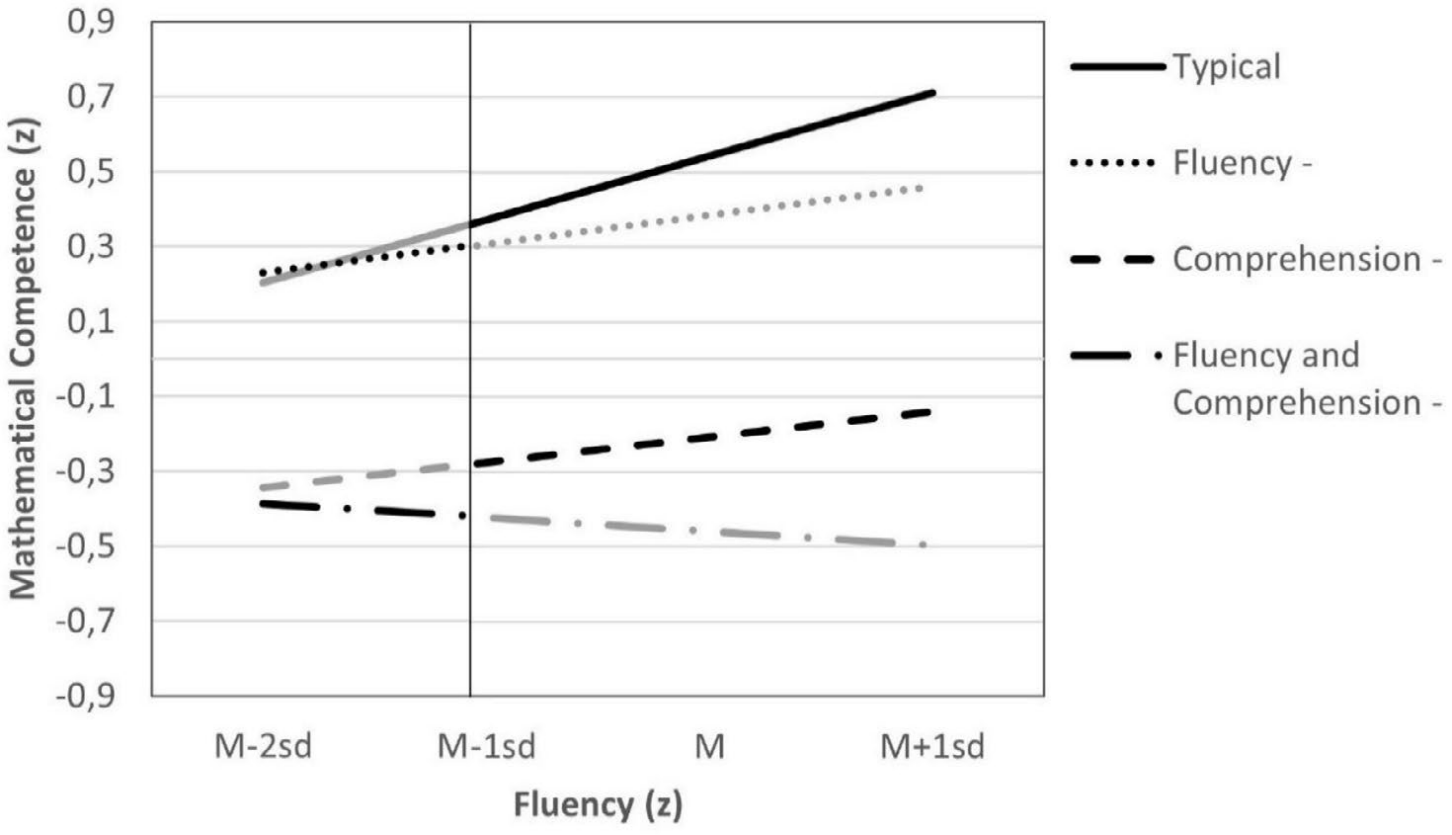
Effects of fluency on mathematical competence in typical readers and students with reading difficulties. *Note:* The black lines are based on the data. The grey lines are based on regression.

To clarify these interactions, a simple slope analysis was conducted (see Table 5). For typical readers, fluency was more strongly linked to mathematical competence than for students with reading difficulties. Hence, the fluency‐math association differs between the groups. In all groups with reading difficulties, fluency is associated positively with mathematical competence. This main effect is qualified by group for students with difficulties in reading comprehension only. For students with difficulties in both skills, the interaction and the main effect of fluency taken together result in a slightly negative association between fluency and mathematical competence. Figure 1 illustrates this picture. In both groups with difficulties in reading comprehension, the main effect of group indicates a poorer mathematical competence than the other groups.

**TABLE 5:** Simple slope analyses for fluency.

Effect	*B*	SE	95% CI		*t*
			LL	UL	
Typical readers					
Fluency	−0.02	0.03	−0.08	0.03	−0.79
Typical reader vs. rest	0.70	0.04	0.63	0.77	19.67***
Typical readers × fluency	0.19	0.03	0.13	0.25	6.26***
Difficulties in fluency only					
Fluency	0.23	0.01	0.21	0.26	18.34***
Fluency only vs. rest	−0.09	−0.02	−0.41	0.23	−0.55
Fluency only × fluency	−0.16	0.12	−0.39	0.07	−1.35
Difficulties in comprehension only					
Fluency	0.23	0.01	0.21	0.26	19.56***
Comprehension only vs. rest	−0.71	0.04	−0.78	−0.63	−18.29***
Comprehension only × fluency	−0.17	0.04	−0.25	−0.08	−3.87***
Difficulties in both					
Fluency	0.18	0.01	0.16	0.21	15.03***
Difficulties in both vs. rest	−0.96	0.23	−1.40	−0.51	−4.22***
Difficulties in both × fluency	−0.22	0.16	−0.53	0.09	−1.40

Abbreviations: CI = confidence interval, LL = lower limit, UL = upper limit.

*** *p* < 0.001.

These results were confirmed when differences between groups at a high (one SD above the mean) and low level (two and one SD below the mean) of fluency were probed. At a high level of fluency, typical readers outperformed students with reading disabilities, especially students with difficulties in reading comprehension. For students with difficulties in fluency or comprehension, the impact of fluency on mathematical competence is a bit weaker than for typical readers. For students with difficulties in both fluency and comprehension, faster reading is not associated with better mathematical competence. Note that the groups do not comprise the whole spectrum of fluency, as the groups were built by using the results in fluency (see black and grey lines in Figure 1).

### 3.2 Impact of Reading Comprehension on Mathematical Competence

The last step was building a regression model for the impact of reading comprehension on mathematical competence. As can be seen in Table 6, the main effects of reasoning and comprehension were significant. In all groups, good reasoning and reading comprehension are associated with better mathematical competence. Only one of the interactions and no group effects were found to be significant.

**TABLE 6:** Moderated regression analysis for the effects of group and comprehension on mathematical competence.

Effect	*B*	SE	95% CI		*t*
			LL	UL	
Intercept	−0.05	0.04	−0.13	0.03	
Reasoning^a^	0.31	0.01	0.28	0.33	27.35***
Comprehension^a^	0.47	0.03	0.42	0.53	16.05***
Typical reader vs. students with reading difficulties^b^	0.12	0.06	−0.01	0.24	1.83+
Students with difficulties in fluency vs. difficulties in reading comprehension^c^	−0.19	0.23	−0.63	0.26	−0.81
Students with difficulties in fluency only vs. difficulties in fluency and comprehension^d^	−0.27	0.15	−0.57	0.03	−1.74+
Typical readers vs. students with reading difficulties × comprehension	−0.07	0.05	−0.16	0.02	−1.58
Students with difficulties in fluency vs. difficulties in reading comprehension × comprehension	−0.13	0.14	−0.40	0.14	−0.95
Students with difficulties in fluency only vs. difficulties in fluency and comprehension × comprehension	−0.19	0.10	−0.38	−0.005	−2.02*
Model fit	*R* ^2^ = 0.48				
Omnibus test	*F* (8, 5009) = 585.79, *p* < 0.001				

Abbreviations: CI = confidence interval, LL = lower limit, UL = upper limit.

^a^ *z*‐Standardised. Coefficients can be interpreted like standardised betas.

^b^ Contrast‐coded: typical readers = 0.75, students with reading difficulties = −0.25.

^c^ Contrast‐coded: students with difficulties in fluency only = −1/2, students with difficulties in reading comprehension = 1/2.

^d^ Contrast‐coded: students with difficulties in fluency only = 1/2, students with difficulties in reading comprehension and fluency = −1/2.

^+^ *p* < 0.10.

* *p* < 0.05.

*** *p* < 0.001.

To clarify this interaction, a simple slope analysis was conducted (see Table 7). Comprehension is positively linked to mathematical competence in all groups. However, for students who have difficulties in both skills, this link is weaker than in the other groups. The groups differ in their level of mathematical competence. It is striking that for students with difficulties in reading speed only, the impact of comprehension on mathematical competence (not the level) is similar to the other groups. It is possible that this is a result of them compensating for their difficulties.

**TABLE 7:** Simple slope analyses for reading comprehension.

Effect	*B*	SE	95% CI		*t*
			LL	UL	
Typical readers					
Reading comprehension	0.43	0.03	0.37	0.48	16.07***
Typical reader vs. rest	0.22	0.04	0.14	0.29	5.81***
Typical readers × comprehension	0.04	0.03	−0.02	0.09	1.20
Difficulties in fluency only					
Reading comprehension	0.49	0.01	0.47	0.52	43.11***
Fluency only vs. rest	−0.19	0.04	−0.27	−0.11	−4.75***
Fluency only × comprehension	−0.02	0.06	−0.12	0.09	−0.28
Difficulties in comprehension only					
Reading comprehension	0.49	0.01	0.47	0.52	38.89***
Comprehension only vs. rest	−0.22	0.14	−0.49	0.05	−1.61
Comprehension only × comprehension	−0.13	0.09	−0.32	0.05	−1.44
Difficulties in both					
Reading comprehension	0.48	0.01	0.46	0.50	40.07***
Difficulties in both vs. rest	−0.61	0.19	−0.98	−0.24	3.20**
Difficulties in both × comprehension	−0.25	0.11	−0.46	−0.03	−2.23*

Abbreviations: CI = confidence interval, LL = lower limit, UL = upper limit.

* *p* < 0.05.

** *p* < 0.01.

*** *p* < 0.001.

**FIGURE 2:**
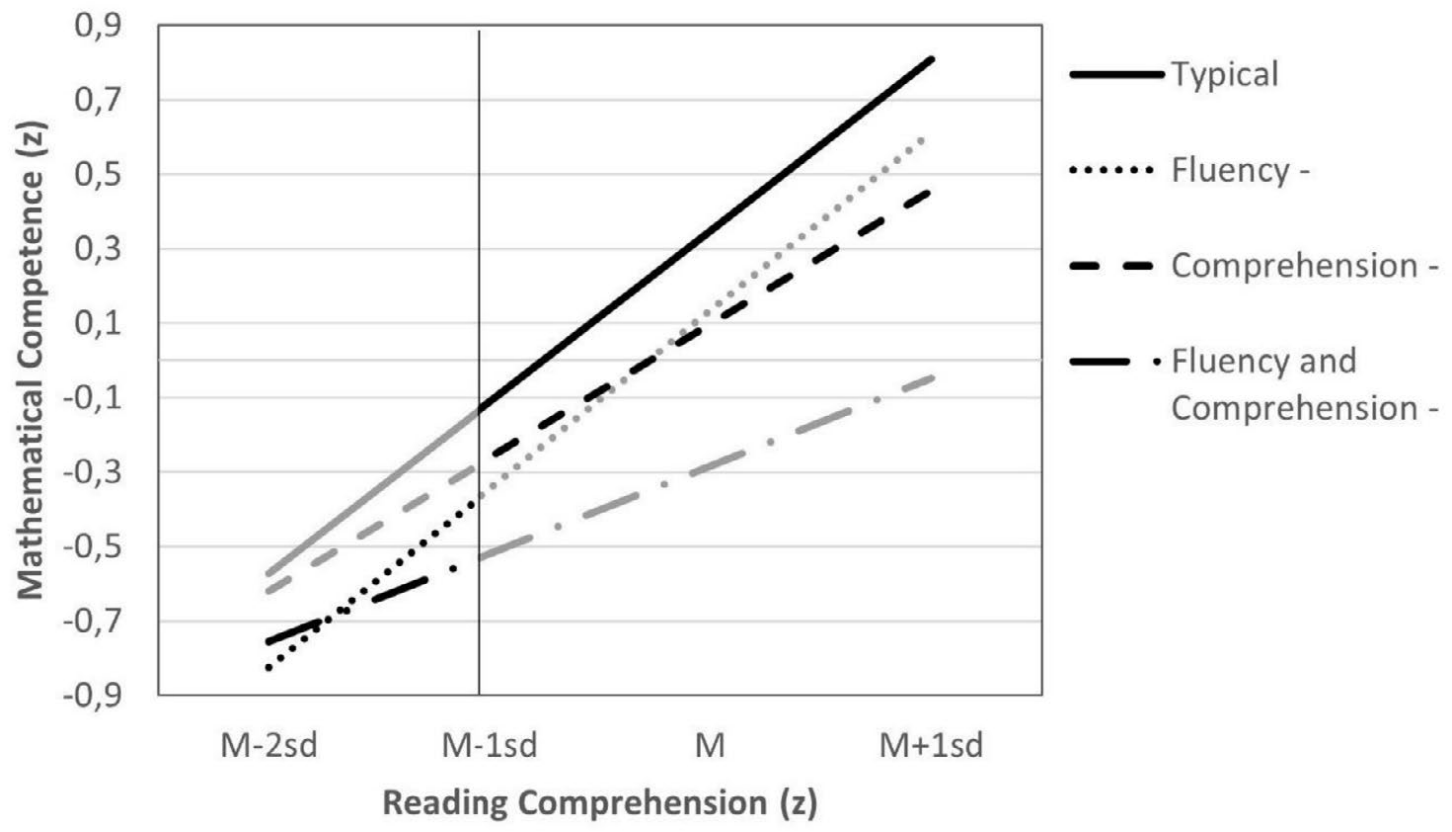
Effects of reading comprehension on mathematical competence in typical readers and students with reading difficulties. *Note:* The black lines are based on the data. The grey lines are based on regression.

These results become evident when differences between groups at a high (one SD above the mean) and low level (two and one SD below the mean) of reading comprehension are considered (see Figure 2). For students with difficulties in fluency only, reading comprehension had a similar impact on word problem solving as it did on typical readers, though their scores were at a lower level. Even for students with difficulties in both skills, better comprehension is associated with better mathematical competence.

## 4 Discussion

The aim of this study was to clarify how fluency and comprehension affect word problem solving in mathematics. The impact of fluency on word problem solving has not been studied yet, but prior research has shown that reading comprehension influences word problem solving even when word problems are read out loud. In the present study, the word problems were read silently by the students themselves. Based on the existing research, it was hypothesised that both fluency and comprehension should affect word problem solving.

The results of this study extend earlier research on the impact of reading comprehension on word problem solving by incorporating fluency. As expected, reading comprehension had an impact on mathematical word problem solving when the problems were not read out loud. The better students understood a text they had read, the better they performed in word problem solving. Fluency also contributed to word problem solving. The faster students could read, the better they performed in word problem solving. Furthermore, students who struggled with comprehension performed better when their reading speed was high and vice versa, students with poor fluency could compensate with strong comprehension.

Overall, reading comprehension had a stronger influence on word problem solving than reading fluency (shown by a steeper slope). This effect was most apparent in students with poor comprehension and fluency. For these students, reading comprehension still helped in word problem solving, albeit less than it did for typical readers or those with only one reading deficit. Fluency, however, had no significant impact on word problem solving, despite a slightly negative slope. This indicates that reading speed did not contribute to word problem solving.

To be clear, even when only one of the reading skills was impaired, students obtained lower mathematical competence scores than typical readers. Moreover, if only one skill was impaired, that skill did not have a greater impact on word problem solving than the same skill had for students who did not have that impairment. However, if fluency and comprehension were both impaired, then those skills contributed less to word problem solving than they did for typical readers or those with only one impaired skill.

The different levels of word problem solving in students with and without reading difficulties are in line with the MDM and other studies (Pimperton and Nation 2010; Träff and Passolunghi 2015; Vukovic et al. 2010). Moreover, the comparable influence of reading fluency and comprehension across varying proficiency levels also aligns with MDM predictions. Fluency and word problem solving seem to have a shared cognitive skill, as do reading comprehension and word problem solving. The results also indicate, however, that students who struggle with both fluency and comprehension appear to have a larger or qualitatively different cognitive deficit than the other groups. In this group, fluency and comprehension do not have the same impact on word problem solving than in the other groups. In the framework of the MDM, this suggests that the group possesses special cognitive characteristics, such as an additional or different cognitive deficit.

Simply put, fluency affects word problem solving. Faster readers can solve more word problems in a given time frame. Reading comprehension has an even stronger influence and better comprehension skills are more helpful in solving word problems than fluency. Students in whom both skills are impaired have the most difficulty solving word problems and only benefit from better comprehension and not from reading faster.

Students who struggle with both reading skills are most likely to receive a dyslexia diagnosis. Dyslexia frequently co‐occurs with dyscalculia. Therefore, it cannot be ruled out that the present results were influenced by dyscalculia. It is estimated that between 25% (van Bergen et al. 2025) and 40% (Wilson et al. 2015) of individuals with dyslexia also have dyscalculia. Consequently, most participants in the present study are unlikely to have had dyscalculia and its overall findings on word problem solving are unlikely to be the result of dyscalculia.

Fluency is necessary to facilitate comprehension (Hoover and Gough 1990; Klauda and Guthrie 2008; LaBerge and Samuels 1974). The results indicate that comprehension has a greater impact on word problem solving than fluency. This may be because most students possessed adequate fluency, so the influence of fluency is less evident. It is also possible that slow readers compensate for their slow reading by skimming texts. This may produce an inadequate situational model, resulting in weak reading comprehension and the wrong word problem solutions.

One striking point merits closer examination. Only 27% of the students with less than average fluency and comprehension (not caused by cognitive impairments) also have a reported dyslexia diagnosis. These students should be quite conspicuous at school and when doing homework. Further research should consider factors such as SES, migration background or private tutoring to determine if they cause educators to overlook their difficulties in reading. This finding also underscores the need for regular, standardised diagnostic screening in schools to ensure that students with reading difficulties are not overlooked.

### 4.1 Limitations and Implications

The reading abilities of fifth‐graders were examined using the NEPS data, yielding several avenues for future research. For example: Because the NEPS also includes students from other grades, the findings could be extended to reading, mathematics and dyslexia across additional grade levels. Results from this study only apply for fifth‐graders.

The role of fluency in orthographies beyond German remains unexplored. As the fluency deficits affect students with dyslexia in both consistent and inconsistent orthographies (e. g., German and English), comparable results are likely in other languages. Future research should address this question.

A methodological issue arises in the classification of students with reading difficulties into three groups: students with difficulties in fluency, students with difficulties in comprehension and students with difficulties in both. Students were assigned to these groups if their fluency or comprehension was below average, but their reasoning score was at least average. As a result, the variance in fluency and comprehension scores is limited, which may produce less precise parameter estimations in each group.

The clustered structure of the data offers another possible direction for future research. Individual classes, schools and states are well‐known to differ in their learning outcomes, so reading and math achievement levels likely vary across the classes included in this study. This paper, however, focused on the effect of reading skills on word problem solving, not on absolute achievement levels. It assumed that this impact of reading on word problem solving is similar across classes. Empirical tests of this assumption are needed in subsequent studies.

In a broader perspective, the findings of this study suggest that measuring mathematical competence with word problems may not yield a valid measurement of mathematical competence, because non‐mathematical deficits (e.g., reading difficulties) affect the scores. Future research should determine whether specific items are more vulnerable to fluency or comprehension deficits than others.

Students with dyslexia will certainly need accommodations to compensate for their reading difficulties when confronted with word problems. Teachers might consider modifying instructions for tasks in which reading is not meant to be part of the resulting measure. One straightforward strategy could be to simply read instructions and word problems out loud. This should help all students to construct a situational model, though the studies mentioned in the first section of this paper reported that students with dyslexia still score lower in word problem solving even when texts were read aloud.

Other accommodations for students diagnosed with dyslexia could include extended testing time for low fluency or assistive tools (e.g., reading pens, text‐to‐speech‐software) for poor comprehension. Future research should then consider how these accommodations affect performance on word problem solving in students who are weaker in these areas.

Finally, schools and educators should be aware that word problems may be an unsuitable measure of competence in mathematics. This observation may well extend to other subjects such as biology, history, chemistry or any domain that uses word problems in assessments.

## Funding

The author has nothing to report.

## Ethics Statement

The data were collected considering all ethical standards and parental consent.

## Conflicts of Interest

The author declares no conflicts of interest.

## Data Availability

Data are available as a scientific use file after signing a data use agreement (doi:10.5157/NEPS:SC3:11.0.1).

## Endnotes

^1^
According to the definition of dyslexia in the DSM‐V (‘specific learning disability with impairment in reading’), the disorder is excluded if IQ < 70. In the ICD‐11, dyslexia (‘developmental learning disorder with impairment in reading’) is characterised by a discrepancy between reading performance and the level of intellectual function.

^2^
DSM‐V points out that *T* < 30 might indicate specific learning disability (dyslexia).
